# Rhizosphere Microbial Community Diversity and Function Analysis of Cut Chrysanthemum During Continuous Monocropping

**DOI:** 10.3389/fmicb.2022.801546

**Published:** 2022-03-16

**Authors:** Tan Wang, Kexin Yang, Qingyun Ma, Xu Jiang, Yiqing Zhou, Delong Kong, Zhiye Wang, Rebecca E. Parales, Lin Li, Xin Zhao, Zhiyong Ruan

**Affiliations:** ^1^CAAS-CIAT Joint Laboratory in Advanced Technologies for Sustainable Agriculture, Institute of Agricultural Resources and Regional Planning, Chinese Academy of Agricultural Sciences, Beijing, China; ^2^State Key Laboratory of Agricultural Microbiology, Huazhong Agricultural University, Wuhan, China; ^3^Institute of Vegetables and Flowers, Chinese Academy of Agricultural Sciences, Beijing, China; ^4^Key Laboratory of Microbial Resources Exploitation and Application of Gansu Province, Institute of Biology, Gansu Academy of Sciences, Lanzhou, China; ^5^Department of Microbiology and Molecular Genetics, College of Biological Sciences, University of California, Davis, Davis, CA, United States; ^6^College of Resources and Environment, Tibet Agricultural and Animal Husbandry University, Linzhi, China; ^7^College of Life Sciences, Yantai University, Yantai, China

**Keywords:** cut chrysanthemum, rhizosphere, community structure, soil physical and chemical property decline, continuous cropping barrier

## Abstract

As an ornamental flower crop, the long-term continuous monocropping of cut chrysanthemum causes frequent occurrence of diseases, seriously affecting the quality of cut chrysanthemum. The rhizosphere microbial community plays an important role in maintaining the healthy growth of plants, whereas the composition and dynamics of rhizosphere microbial community under continuous monocropping of cut chrysanthemum have not been fully revealed. In this study, the Illumina MiSeq high-throughput sequencing platform was used to monitor the dynamic changes of rhizosphere microbial communities in four varieties of cut chrysanthemum during 0–3 years of monocropping, and the soil physicochemical properties were also determined. Results showed that continuous monocropping significantly increased the fungal community richness and altered the profiles of the bacterial and fungal communities, leading to variation of community beta-diversity. With the increase of continuous cropping time, biocontrol bacteria decreased, while some plant pathogenic fungi were enriched in the rhizosphere of cut chrysanthemum. FAPROTAX-based functional prediction showed that the abundance of gene related to nitrogen and sulfur metabolism and chitin lysis was reduced in the rhizosphere of cut chrysanthemum. FUNGuild-based fungal function prediction showed that plant pathogenic fungal taxa were increasing in the rhizosphere of cut chrysanthemum, mainly *Acremonium*, *Plectosphaerellaceae*, *Fusarium*, and *Cladosporium*. Continuous cropping also reduced the content of ammonium nitrogen and increased soil salinity, resulting in deterioration of soil physical and chemical properties, which, together with the transformation of rhizosphere microbial community, became part of the reasons for the continuous cropping obstacle of cut chrysanthemum.

## Introduction

Cut chrysanthemum is a popular flower crop because of its easy breeding, long bottle life, and storage transport. It is also known as a major cut flower worldwide, and its main production areas cover Netherlands, China, Japan, and Korea (Li et al., 2016). In China, the planting area of cut chrysanthemum exceeds 7,000 hectares, and the annual sales reach 2.65 billion sprays with a sales volume of 1.52 billion yuan (China Ministry of Agriculture, 2016). Cut chrysanthemum is generally grown in greenhouse sheds, which can be harvested up to five times a year, to obtain great economic benefits. However, similar to other crops, such as soybean, tomato, watermelon, and vanilla, long-term continuous monocropping will generate replanting problems. The replanting problem of cut chrysanthemum will induce adverse symptoms such as short stature, yellowing, and increased infection rate (Li et al., 2017). In commercial production, the replanting problem has led to a significant decline in the yield and quality of cut chrysanthemum, resulting in great economic losses for growers.

At present, a number of measures to address the replanting problem have been developed. Rotation can reduce weed and pest occurrence, facilitate soil nutrient utilization (Nevens and Reheul, 2001), and improve crop yield and quality (Zhao et al., 2020). Organic fertilizer contains a variety of organic acids (OA), peptides, and rich nutrient elements such as nitrogen, phosphorus, and potassium, which can not only provide comprehensive nutriment for crops but also increase and renew soil organic matter, promote microbial reproduction, and improve soil physical and chemical properties and biological activity (Chen et al., 2018). Long-term continuous monocropping will cause the accumulation of pathogenic microorganisms, and the application of soil sterilization (including high-temperature sterilization and reagent fumigation) can effectively reduce the number of pathogenic microorganisms (Li et al., 2019). However, soil sterilization may damage the soil microbial ecology, which is contrary to the objective of sustainable agriculture. Rhizosphere microbial communities are closely related to plant health (Raaijmakers et al., 2009). The root exudates from plants consist of phenolic acids (PA), OA, sugars, amino acids, fatty acids, alkaloids, terpenoids, enzymes, flavonoids, and proteins (De Weert et al., 2002; Bais et al., 2006). A variety of carbon and nitrogen sources obtain abundant microorganisms. Exploiting microorganisms isolated from the environment as antagonists or growth-promoting rhizobacteria can effectively reduce the occurrence of diseases and promote plant growth (Verbon and Liberman, 2016; Morales-Cedeño et al., 2021).

Nevertheless, the aforementioned method always transforms the soil microbiome to a healthier direction. As the second genome of plants, plant-associated microbiome plays an important role in plant growth. With regard to nutrient utilization, the symbiotic interactions between plants and arbuscular mycorrhizal fungi and *Rhizobium* bacteria lead to nutrient acquisition (Richardson and Simpson, 2011; Corné et al., 2014; Trivedi et al., 2016; Rachel et al., 2018). Non-symbiotic plant-growth-promoting bacteria can improve not only the bioavailability of insoluble minerals but also the root system architecture of host plants, thereby increasing the exploratory capacity of the root for water and minerals (Richardson and Simpson, 2011; Trivedi et al., 2016). A recent study suggested that the differences in nitrogen use efficiency in rice varieties are due to a high proportion of nitrogen-cycling-related bacterial supplementation, resulting in a more efficient transformation of nitrogen in the root environment in indica rice than in japonica rice varieties (Zhang et al., 2019). Rhizosphere microorganisms extend the bioavailability of nitrogen by nitrification, delay flowering time by converting tryptophan to the plant hormone indoleacetic acid, and stimulate plant growth by downregulating flowering genes (Lu et al., 2018). Plant-related microbial groups can mobilize nutrients that are not easily available to plants, such as inorganic phosphate and iron, through dissolution, mineralization, or excretion through iron-chelated siderophores. Moreover, plant-related microbiome helps plants fight diseases. The effect of plant defense based on natural microorganisms on plant health has been clearly demonstrated in suppressive soils. Plant root exudates stimulate, enrich, and support soil microorganisms as the first line of defense against soil-borne pathogens (Mendes et al., 2011). Community-based analyses of suppressive soils have demonstrated that no single phylum is uniquely associated with disease suppression (Mendes et al., 2011; Penton et al., 2014; Santhanam et al., 2015; Cha et al., 2016; Trivedi et al., 2017). A recent study has modeled general disease suppression of *Fusarium oxysporum* and identified bacterial abundance in *Actinomycetes* and *Firmicutes* as predictive markers for continent-scale soil disease suppression. Under abiotic or biological stress conditions, plants usually select a microbial community that promotes stress resistance (Wagner et al., 2014; Eida et al., 2018; Naylor and Coleman-Derr, 2018; Naylor et al., 2018; Timm et al., 2018; Fitzpatrick et al., 2019), which modifies plant evolutionary responses to environmental stress by altering the fitness of individual plant genotypes, the expression of plant traits related to fitness, and the strength or direction of natural selection occurring within populations that experience environmental stress through the effects of microorganisms on reproductive fitness (Lau and Lennon, 2012; Eida et al., 2018; Fitzpatrick et al., 2018, 2019). In the effect of fungal endophytes on plant growth performance under water stress, traits related to resource utilization and stress tolerance account for 26–53% (Kudjordjie et al., 2019). In addition, the complex microbial community can help plants deal with other adverse effects, such as heavy metals (Yang et al., 2020), herbicides (Fawzy et al., 2015), and pesticides (Li et al., 2020). The composition and structure of the microbiome must remain stable to continuously exert the functional potential of the plant-related microbiome, and the loss of its steady state may cause negative effects such as imbalance in soil nutrient conversion and the proliferation of pathogenic bacteria. Therefore, determining the dynamic changes of the rhizosphere microbial community under different continuous cropping periods is necessary to explore the mechanism of continuous cropping obstacles of cut chrysanthemum.

Based on previous studies, the rhizosphere microbial community of cut chrysanthemum is based on denaturing gradient gel electrophoresis (Duineveld et al., 2001; Song et al., 2013), which is not sufficient to reveal the overall dynamic changes of bacteria and fungi during continuous cropping of cut chrysanthemum. With the wide application of high-throughput sequencing technology, the microbial community landscape in the environment is gradually revealed. Here, we used Illumina MiSeq sequencing technology to detect the dynamic changes of microbial communities in the rhizosphere soil of four varieties of cut chrysanthemum during 0–3 years of monocropping period and measured the physical and chemical properties, nutrients, and other indicators of soil to evaluate the health status of the soil during continuous cropping. We hypothesized that (i) during continuous monocropping for 0–3 years, harmful microorganisms would accumulate in the rhizosphere. (ii) Differences in rhizosphere exudates of different varieties of cut chrysanthemum could affect the rhizosphere microbial community. (iii) With the increase of continuous monocropping time, soil physicochemical properties decline, and nutrient conversion slows down.

## Materials and Methods

### Study Site and Experimental Design

The experimental site was located in Nankou experimental base of the Chinese Academy of Agricultural Sciences, Beijing (116.57033, 40.237361), China. Cut chrysanthemums with different monocropping times were planted in different greenhouses at a distance of 8 m from each other. Each greenhouse is 80 m in length. In the greenhouse, each variety of cut chrysanthemum was randomly planted in three plots, separated by other cut chrysanthemum varieties. All plots adopted the same water and fertilizer management practices. During sampling, each plot randomly took soil samples of three chrysanthemum plants and mixed them into one replicate. The monoculture time selected in the experiment was 0 years (CK, before planting cut chrysanthemum), 0.5 years (H, planting for one crop), and 3 years (T, planting for six crops). The sampling period was the mature period. A total of four varieties were selected, namely, *Glitter Orange* (variety 1), *Gem Yellow* (variety 2), *Cloud Red* (variety 3), and *Mona Lisa Pink* (variety 4). Thus, the sample names were CK (0 years); H1, H2, H3, and H4 (0.5 years); and T1, T2, T3, and T4 (3 years). CK had four replicates; the others had three replicates. In obtaining rhizosphere soil, the fibrous roots and main roots of the plant were cut off with sterile scissors, and then the roots were placed into a sterilized 50 ml centrifuge tube. Subsequently, the root tissues were added with 0.2 × PBS buffer solution and swirled for 1 min. Afterward, the root tissues were removed and centrifuged at 8,000 rpm for 5 min. The supernate was discarded, and the residual part was the rhizosphere soil. The rhizosphere soil samples were placed at –80°C for subsequent DNA extraction.

### Soil Physicochemical Properties

The planted soil of cut chrysanthemum (bulk soil) was collected to determine the physical and chemical properties. The sampling method of bulk soil referred to that of Edwards et al. (2014). The collected soil was air-dried on a kraft paper and screened by a 1 mm sieve, and the resulting soil was determined as dried soil. Soil pH was measured using a glass conductance meter in a 1:2.5 soil/water (W/V) suspension (Qiu et al., 2012). The content of soil total carbon, total nitrogen, and organic carbon was determined by using an elemental analyzer (Elementar Analysensysteme GmbH, Germany). Soil urease (Kandeler and Gerber, 1988), catalase (Johansson, 1988), sucrase (Gao et al., 2013), and alkaline phosphatase (Scandinavica et al., 2014) activities were determined using the soil enzyme activity assay kit from Solarbio Science & Technology Co. (Beijing, China). Soil available P was extracted using sodium bicarbonate and measured with the molybdenum blue method (Olsen, 1954). Soil available K was extracted using ammonium acetate and determined with flame photometry (nova 300, Analytic Jena, Germany) (Frank et al., 1998). Soil ammonium nitrogen and nitrate nitrogen were extracted with 1 M KCl and the indophenol-blue colorimetric and double wavelength (220 and 275 nm) methods, respectively, and their concentrations were measured using a spectrophotometer (UV-6000, China) (Zhao et al., 2017). Soil salinity was determined by an oven-drying method following the NY/T 1121.16-2006 standard water-soluble salt analysis of soil.

### Soil DNA Extraction and High-Throughput Amplicon Sequencing

Total genome DNA from 0.5 g of fresh soil samples was extracted using a E.Z.N.A™ Mag-Bind Soil DNA Kit (OMEGA, Norcross, United States) following the manufacturer’s instructions. DNA integrity was detected on 1% agarose gel, and DNA concentration was quantitatively detected by Qubit. The V3–V4 region of the 16S rRNA gene was amplified using the PCR primers 341F (5′-CCTACGGGNGGCWGCAG-3′) and 805R (5′-GACTACHVGGGTATCTAATCC-3′) for bacterial community analysis, and the ITS1 region of the transcribed spacer was amplified using the PCR primers ITS1F (5′-CTTGGTCATTTA GAGGAAGTAA-3′) and ITS2R (5′-GCTGCGTTCTTCATCGATGC-3′). 16S rRNA genes were amplified using a specific primer with a barcode. The first round of PCRs was performed in 30 μl of reactions with 15 μl of 2 × Hieff Robust PCR Master Mix, 1 μl of Bar-PCR primer F and 1 μl of Primer R, and 10–20 ng of template DNA. PCR amplification cycles consisted of an initial denaturation at 94°C for 3 min, followed by 5 cycles of denaturation at 94°C for 30 s, annealing at 45°C for 20 s, and elongation at 65°C for 30 s; then 20 cycles of denaturation at 94°C for 20 s, annealing at 55°C for 20 s, and elongation at 72°C for 30 s; and finally elongation at 72°C for 5 min after cycles.

Illumina bridge PCR-compatible primers were introduced in the second round of amplification. The reaction system was identical to that of the first round; however, the primers were replaced by Primer F and Index-PCR Primer R. Library quality was assessed using a Qubit@ 3.0 fluorometer (Thermo Scientific, Waltham, MA, United States). Finally, the library was sequenced on an Illumina MiSeq platform, and 250 bp paired-end reads were generated.

### Community Diversity and Function Analysis

USearch was used to cluster the sequences in the libraries. Operational taxonomic unit (OTU) clustering was performed on non-repeated sequences (excluding single sequences) based on 97% similarity, and the representative sequence of OTU was obtained by removing chimeras during clustering. Then, an RDP classifier was used to annotate taxonomic information for each representative sequence. In calculating alpha diversity, we refined the OTU table and calculated two indicators, namely, ACE index and Shannon even index, to estimate the species richness and species evenness of the community, respectively.

The 10 most abundant phyla were selected from all the samples included in the phylum-level species annotation and abundance information, and a histogram was drawn for each sample with regard to relative abundance. In addition, 30 genera with the highest abundance were selected to draw a heatmap. LEfSe (Segata et al., 2011) was used to compare data among samples and select biomarkers for each sample, and 2.0 was set as the threshold for the logarithmic LDA score for discriminating features. Principal co-ordinate analysis (PCoA) was performed to compare the community profiles of different samples in R^1^ by using the amplicon and phyloseq packages. Redundancy analysis (RDA) was performed to evaluate the relationship between microbial community profiles and soil physical and chemical properties by using the ggvegan package. Linear fitting analysis was used to evaluate the correlation between soil enzyme activity and soil nutrient element content and between soil enzyme activity and microbial community richness index. To further explore the impact of microbial community change, functional prediction with PICRUSt (Langille et al., 2013), FAPROTAX (Louca et al., 2016), and FUNGuild (Nguyen et al., 2015) annotation tools was performed. The predicted results were visualized by a principal component analysis (PCA) plot and boxplot. The figures were adjusted, combined, and modified by Adobe Illustrator 2021.

## Results

### Changes of Soil Physicochemical Properties of Cut Chrysanthemum During Continuous Monoculture

During continuous monocropping for 0–3 years, the contents of soil total carbon, total nitrogen, and organic carbon increased significantly in soil planted with “variety 1” and “variety 4,” whereas no evident change was observed in soil pH of all varieties (Table 1). Ammonium nitrogen content showed a decreasing trend in bulk soil of all varieties. In general, the content of nitrate nitrogen initially increased and then decreased during continuous monocropping, but it increased remarkably in soil planted with “variety 1” in the third year of monocropping. The content of available phosphorus and potassium also showed an increasing trend in replanted soil, and the increment in bulk soil planted with “variety 1” and “variety 4” was high. Likewise, the total salt content in soil increased in the third year of monocropping, and the maximum increment was observed in soil planted with “variety 1.”

**TABLE 1:** A summary of soil physicochemical properties in bulk soil of cut chrysanthemum.

Treatments	Total carbon (g/kg)	Total nitrogen (g/kg)	Organic carbon (g/kg)	Soil pH	Ammonium nitrogen (mg/kg)	Nitrate nitrogen (mg/kg)	Available phosphorus (mg/kg)	Available potassium (mg/kg)	Salt content (g/kg)
CK	8.84 ± 2.49^d^	0.59 ± 0.14^b^	5.00 ± 0.93^b^	8.63 ± 0.19^a^	1.61 ± 0.25^a^	16.15 ± 4.46^b^	27.77 ± 6.15^ef^	64.80 ± 8.14^f^	0.95 ± 0.09^bc^
H1	8.28 ± 0.16^d^	0.59 ± 0.05^b^	3.84 ± 0.19^b^	8.43 ± 0.16^a^	1.20 ± 0.42^ab^	21.75 ± 7.62^b^	29.84 ± 2.32^de^	87.47 ± 2.47^ef^	0.53 ± 0.09^de^
H2	10.87 ± 0.14^bcd^	0.55 ± 0.07^b^	4.19 ± 0.14^b^	8.53 ± 0.24^a^	1.03 ± 0.34^ab^	19.63 ± 6.50^b^	29.30 ± 3.34^de^	96.13 ± 5.40^ef^	0.64 ± 0.05^cde^
H3	10.18 ± 0.16^cd^	0.56 ± 0.03^b^	4.26 ± 0.24^b^	8.51 ± 0.19^a^	1.07 ± 0.38^ab^	19.45 ± 6.77^b^	34.91 ± 3.71^de^	110.87 ± 0.58^de^	0.87 ± 0.07^bcd^
H4	13.31 ± 0.72^ab^	0.51 ± 0.07^b^	5.06 ± 1.14^b^	8.41 ± 0.05^a^	0.95 ± 0.32^ab^	28.95 ± 4.24^b^	18.17 ± 2.93^f^	76.07 ± 11.5^ef^	1.20 ± 0.06^b^
T1	15.07 ± 0.52^a^	1.19 ± 0.05^a^	10.70 ± 1.09^a^	8.22 ± 0.10^a^	0.54 ± 0.15^b^	65.98 ± 25.27^a^	137.01 ± 3.32^b^	215.17 ± 17.42^b^	1.73 ± 0.05^a^
T2	10.34 ± 0.29^cd^	0.55 ± 0.04^b^	4.77 ± 0.87^b^	8.59 ± 0.25^a^	0.58 ± 0.20^b^	13.26 ± 2.60^b^	39.56 ± 0.92^d^	133.37 ± 0.46^cd^	1.13 ± 0.08^bc^
T3	10.21 ± 0.14^cd^	0.64 ± 0.06^b^	5.13 ± 0.27^b^	8.53 ± 0.07^a^	0.49 ± 0.05^b^	13.66 ± 1.68^b^	58.69 ± 5.41^c^	165.40 ± 13.11^c^	1.04 ± 0.13^bc^
T4	12.62 ± 0.45^abc^	1.17 ± 0.05^a^	10.37 ± 0.53^a^	8.27 ± 0.22^a^	0.81 ± 0.20^ab^	24.37 ± 5.36^b^	150.35 ± 0.97^a^	488.73 ± 21.44^a^	1.05 ± 0.15^bc^

*Statistical significance was set at a level of p < 0.05 using Tukey’s HSD tests. The same letter in the table represents no significant difference.*

**FIGURE 1:**
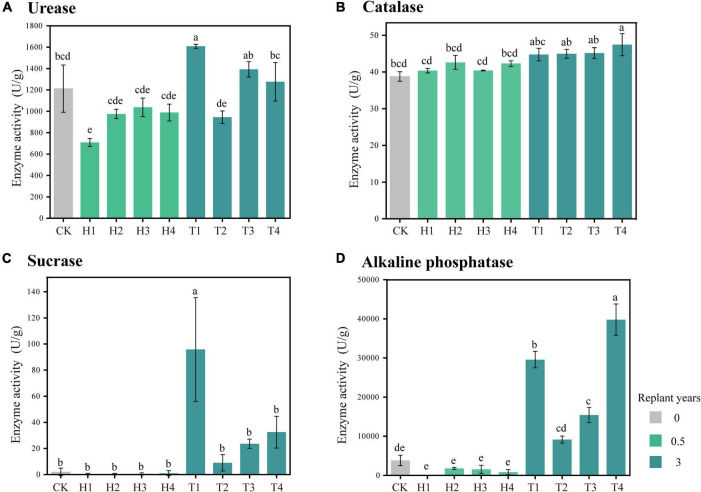
Changes of the activities of four pivotal soil enzymes with the continuous monoculture period (Tukey’s HSD test). **(A)** Urease. **(B)** Catalase. **(C)** Sucrase. **(D)** Alkaline phosphatase.

During monocropping of cut chrysanthemum, the activities of four soil enzymes showed an upward trend in general. Urease activity of soil planted with “variety 1,” “variety 3,” and “variety 4” significantly increased in the third year, and a high increment was observed in bulk soil planted with “variety 1” (Figure 1). The activity of soil catalase also showed an increasing trend with the increase of monocropping time. The activity of sucrase was almost zero from 0 to 0.5 years, but it increased greatly in the third year (T1). Meanwhile, alkaline phosphatase activity increased from 0.5 to 3 years.

### Diversity Changes of Rhizosphere Microbial Community With Continuous Monocropping Period

**FIGURE 2:**
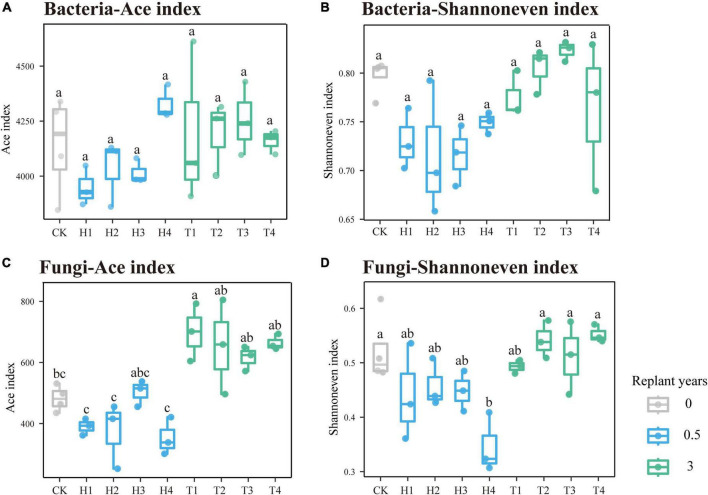
Variations of the rhizosphere microbial community richness and evenness in different monoculture years. The ACE index indicates the richness of community, where the Shannon even index represents the evenness of the community. Box colors show the length of continuous monoculture time. **(A,B)** Prokaryotic community. **(C,D)** Fungal community.

**FIGURE 3:**
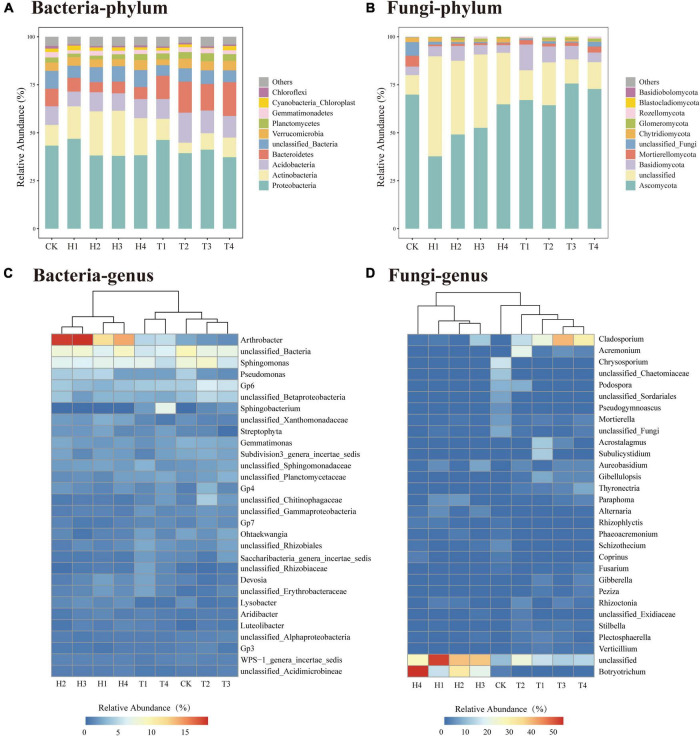
The dynamics of the rhizosphere microbial community composition on phylum and genus levels during the continuous monoculture time series. **(A)** Bacterial community on phylum level. **(B)** Fungal community on phylum level. **(C)** Bacterial community on genus level. **(D)** Fungal community on genus level.

During the continuous monocropping period of 0–3 years, the richness and evenness of the rhizosphere bacterial community of the four varieties of cut chrysanthemum did not change significantly (Figures 2A,B). The richness of the fungal community increased remarkably in the third year, whereas the evenness remained constant (Figures 2C,D). During the monoculture period, the structure and composition of the rhizosphere microbial community changed dramatically. At the phylum level of bacteria (Figure 3A), *Proteobacteria* with the highest relative abundance maintained stable abundance with continuous monocropping. In another dominant phylum, *Actinobacteria*, its abundance decreased significantly in the third year of continuous monocropping, particularly in the rhizosphere of “variety 2.” On the contrary, the abundance of *Bacteroidetes* (Top4 phylum) showed a significant increase in the third year. Furthermore, the abundance of other dominant phyla, such as *Verrucomicrobia*, *Gemmatimonadetes*, and *Chloroflexi*, remained relatively stable in the rhizosphere. In the fungal community (Figure 3B), *Ascomycota* was the most abundant phylum. In year 0.5, the relative abundance of *Ascomycota* varied greatly in the rhizosphere of the four varieties of cut chrysanthemum, but it remained stable in the rhizosphere of the four varieties during the follow-up period. *Basidiomycota* (Top3 phylum) gradually enriched around the root with continuous monocropping, and it showed preference to “variety 1.” The relative abundance of *Mortierellomycota* (Top4 phylum) in CK soil was higher than that in the rhizosphere, and it occupied great proportion in the rhizosphere with continuous monocropping. At the genus level, *Arthrobacter* (Top1 genus) was largely enriched in the rhizosphere of 0.5 year samples, but it declined in the third year (Figure 3C). The relative abundance of *Pseudomonas* (Top4 genus) and *Lysobacter* (Top24 genus) also decreased in the third year of monoculture. *Sphingobacterium* (Top7 genus) was significantly enriched in the rhizosphere of all varieties. Some taxa, such as *Gp6* (Top5 genus), *Streptophyta* (Top9 genus), and *Ohtaekwangia* (Top18 genus), only showed differences in some samples. In the fungal community (Figure 3D), *Cladosporium* (Top1 genus) was greatly enriched in the rhizosphere of cut chrysanthemum with continuous monoculture. *Acremonium* (Top2 genus) and *Thyronectria* (Top14 genus) showed the same trend. An unclassified genus (Top29), and *Botryotrichum* (Top30 genus), showed high abundance in the rhizosphere of cut chrysanthemum in 0.5 years. However, by the third year of monoculture, their abundance dropped dramatically.

**FIGURE 4:**
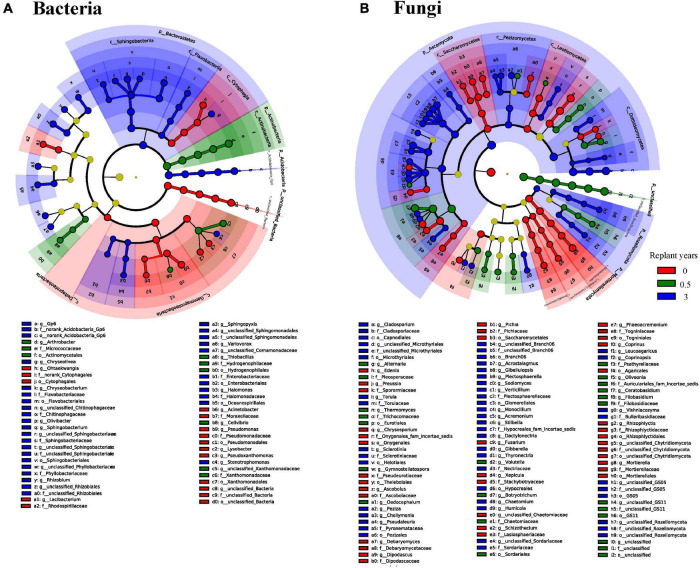
LEfSe of bacterial 16S rDNA **(A)** and fungal ITS rDNA **(B)** sequences with different abundances between continuous monoculture periods. The non-significantly different species are uniformly colored yellow, and the different species biomarkers are colored the same as the groups. The names of species represented by letters are shown in the legend on the bottom.

LEfSe identified the taxa that were significantly different among samples, which were defined as biomarkers (Figure 4). In the bacterial community, the abovementioned bacteria changed significantly in the third year of monoculture, which primarily belonged to *Bacteroidetes* and *Acidobacteria* (Figure 4A). At a low classification level, the families *Sphingobacteriaceae* (Top5 family), *norank_Acidobacteria_Gp4* (Top7 family), *Chitinophagaceae* (Top4 family), *Flavobacteriaceae* (Top25 family), *Planctomycetaceae* (Top9 family), and *Enterobacteriaceae* (Top63 family) and the genera *Sphingobacterium* (Top6 genus), *Gp6* (Top3 genus), *Chryseobacterium* (Top37 genus), *Chryseolinea* (Top29 genus), *Gp7* (Top18 genus), *Sphingopyxis* (Top31 genus), *Pelagibacterium* (Top46 genus), *Rhizobium* (Top40 genus), *Variovorax* (Top33 genus), *Flavobacterium* (Top61 genus), *Olivibacter* (Top63 genus), *Halomonas* (Top51 genus), *Stenotrophomonas* (Top82 genus), *Buttiauxella* (Top106 genus), and *Gp10* (Top35 genus) contributed to the occurrence of differences in the phylum level. For the fungal community, the difference in the rhizosphere community in the third year of monoculturing primarily occurred on *Ascomycota* and *Rozellomycota* (Figure 4B). At the species level, *Cladosporium_sphaerospermum* (Top1 species), *unclassified_Acremonium* (Top3 species), *unclassified_Gibellulopsis* (Top5 species), *Acrostalagmus_luteoalbus* (Top4 species), *Thyronectria rhodochlora* (Top7 species), and *Podospora pyriformis* (Top10 species) had high LDA scores, which partly resulted in the difference at the phylum level.

**FIGURE 5:**
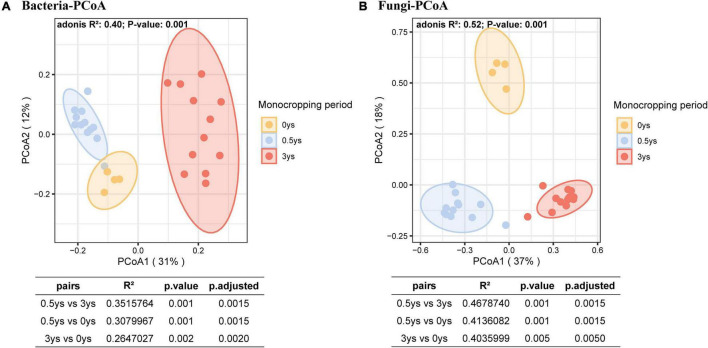
Difference of the rhizosphere microbial community profiles of various monoculture years. Points’ color represents different monoculture periods. The points are covered by a 95% confidence ellipse. Pairwise PERMANOVA is shown below the PCoA plots. **(A)** PCoA of prokaryotic communities. **(B)** PCoA of fungal communities.

**FIGURE 6:**
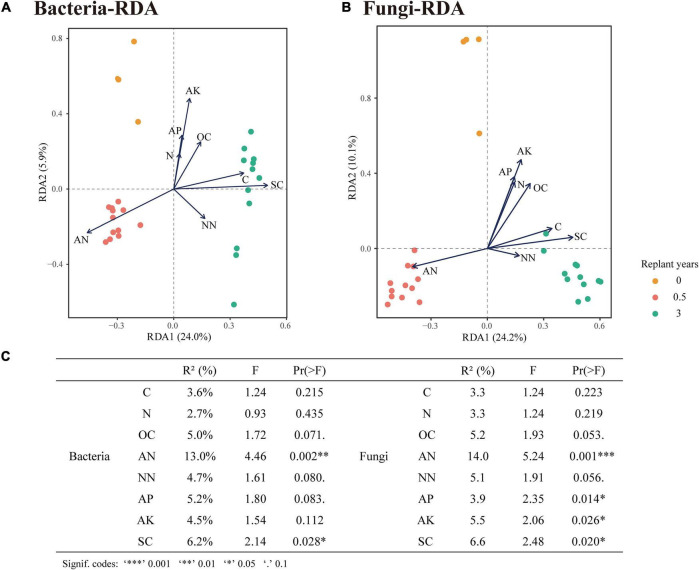
Correlations between soil physicochemical properties and rhizosphere microbial species. **(A,B)** RDA of bacterial and fungal community profiles correlated to soil properties. **(C)** Explanation rate of soil physicochemical properties to the differences of community profiles (conditional effect).

Principal co-ordinate analysis based on the Bray–Curtis distance showed that bacterial or fungal communities of different monocropping periods were separated on the first axis, and the differences accounted for 31% (Figure 5A) and 37% (Figure 5B), respectively. The PERMANOVA test showed significant differences among the three-period samples (*P* < 0.01). RDA of the rhizosphere microbial community and soil physicochemical properties during monocropping showed that (Figures 6A–C) the total contribution rates of soil total carbon (C), total nitrogen (N), organic carbon (OC), ammonium nitrogen (AN), nitrate nitrogen (NN), available phosphorus (AP), available potassium (AK), and soil salinity (SC) in shaping bacterial and fungal communities were 44.9 and 46.9%, respectively, of which the content of ammonium nitrogen had the highest contribution rate (13.0% for bacteria and 14.0% for fungi). On the first axis, the microbial community was primarily separated by AN, C, SC, and NN, whereas on the second axis, the microbial community was separated by OC, N, AP, and AK.

### Functional Prediction of Rhizosphere Microbial Community in Cut Chrysanthemum During Continuous Monocropping

**FIGURE 7:**
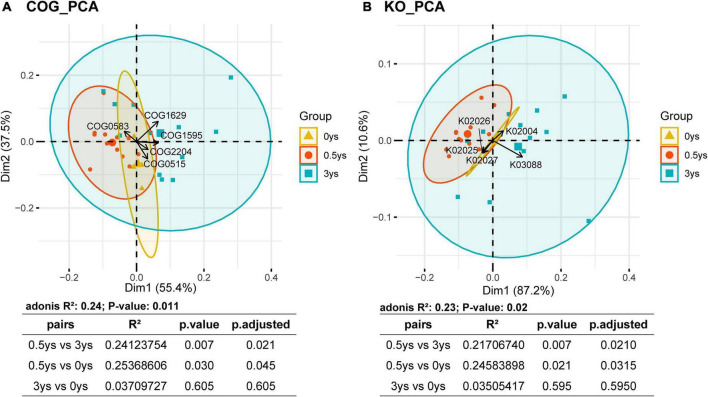
Principal component analysis based on COG **(A)** and KEGG **(B)** functional annotations. The arrows represent the most volatile COG and KEGG classifiers in the functional prediction results. PERMANOVA was used to test the significance of the differences.

Principal component analysis was performed with the results of COG and KEGG function annotations (Figure 7), and the results showed that the functional composition of rhizosphere microorganisms of cut chrysanthemum has changed significantly from 0.5 to 3 years (PERMANOVA, COG: *P* = 0.007, KO: *P* = 0.007). The five COG classifiers with the highest contribution rates that cause differences between samples were COG0583, COG1629, COG1595, COG2204, and COG0515. According to the distribution of sample points of different monocropping years on the PCA plot, it showed that COG1629 was the most influential COG classifier that caused differences of functional profiles. COG1629 was annotated as outer membrane receptor proteins, mostly referring to Fe transport. In the KEGG annotated results, the five most volatile KEGG classifications were K02026, K02004, K03088, K02027, and K02025. The K03088, belonging to RNA polymerase sigma-70 factor, ECF subfamily, had the greatest contribution to differences between groups (0.5 vs. 3 years).

**FIGURE 8:**
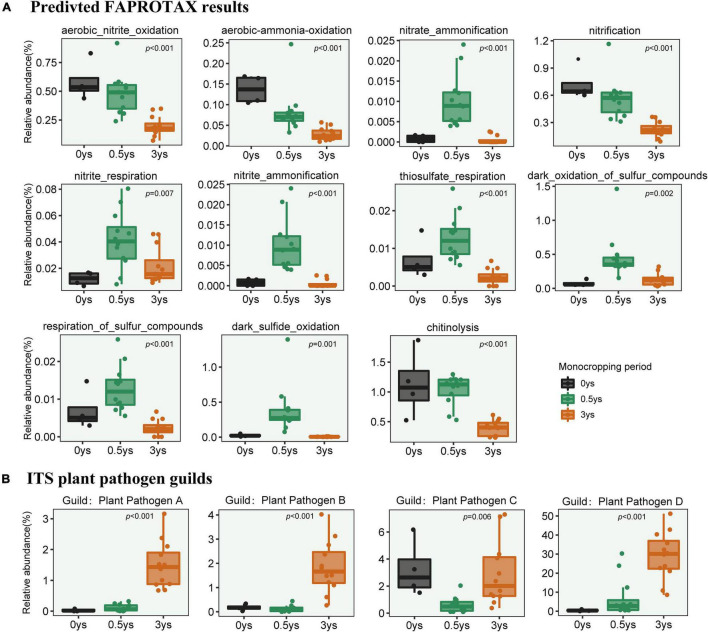
Changes in relative abundance of metabolic pathways in prokaryotic communities based on FAPROTAX **(A)** and variation of abundance of plant pathogen guilds based on FUNGuild **(B)**. ANOVA was used to test the significance of differences.

Based on the results of FAPROTAX and FUNGuild, significant changes in the abundance of some metabolic pathways of bacterial community (Figure 8A) and the plant pathogen of fungal community (Figure 8B) had occurred. Among these pathways, most of them involved nitrogen and sulfur metabolism, and one was associated with chitin lysis. Continuous monocropping obviously reduced the abundance of these metabolic genes. For the fungal community, all species were matched with different functional groups (Guild). Guilds which were part of plant pathogens had been observed in the rhizosphere, and some of them were massively enriched with continuous monocropping.

## Discussion

### Continuous Monocropping Resulted in Soil Degradation of Cut Chrysanthemum

**FIGURE 9:**
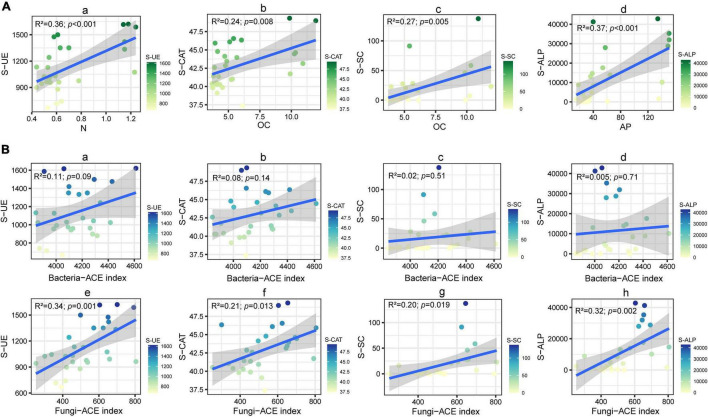
Linear fitting analysis of soil enzyme activity and soil physical and chemical properties **(Aa–Ad)** and microbial community richness index **(Ba–Bh)**. *R*^2^ represented the magnitude of the correlation coefficient, and the *p*-value was generated by a *t*-test.

Here, we monitored the changes of soil-related physical and chemical properties of four varieties of cut chrysanthemum after continuous monocropping for 3 years, and the results showed that the content of soil carbon, nitrogen, available phosphorus, and available potassium showed different degrees of increment in the third year of monocropping (Table 1). The soil pH did not change dramatically, but a slight reduction occurred in the 3 year soil planted with “variety 1” and “variety 4.” Notably, the content of available nitrogen (including ammonium nitrogen and nitrate nitrogen) in the soil basically decreased with continuous cropping, which may cause nutrient imbalance and hinder the growth of cut chrysanthemum. In this study, the salt content in the soil showed an increasing trend with continuous monocropping. Soil salinity is also closely related to the growth of plants. Higher soil salinity produces higher osmotic pressure, which leads to the inability of crops to effectively absorb water and nutrients in the soil (Kluitenberg and Biggar, 1992; Tian et al., 2020), resulting in vegetable deficiency symptoms and decreasing crop quality and yield (Riaz et al., 2018). Soil enzyme, which is produced by plants, animals, and microorganisms, participates in the synthesis and oxygenolysis of various organic and inorganic substances, and it is an indicator for evaluating soil fertility (Dick and Kandeler, 2004; Wang et al., 2019). Soil urease (S-UE) is an enzyme that hydrolyzes amide organic nitrides into inorganic nitrides that can be directly utilized by plants (Singh and Ghoshal, 2013; Cai et al., 2015), while high urease activity rapidly hydrolyzes applied urea to ammonia which contributes to soil nitrogen losses and reduces N use efficiency of crop plants (Rana et al., 2021). Furthermore, ammonium accumulation in soil, particularly at high pH, may hinder the nitrification process at the midway and result in the accumulation of toxic levels of nitrites (Breuillin-sessoms et al., 2017). Soil catalase (S-CAT) is an important oxidoreductase, which is closely related to the activity of oxygen-consuming microbes. It can promote the decomposition of hydrogen peroxide—a metabolic intermediate—and alleviate its toxicity (Sun et al., 2013). The enzymatic activity of catalase can characterize the strength of soil humus and the conversion rate of organic matter (Lee et al., 2009). The soil sucrase activity (S-SC) reflects the maturation degree and fertility level of soil and is an important index for evaluating soil fertility (Stępniewska et al., 2009). Further, sucrase activity is directly related to the growth of crops and closely related to the transformation of organic matter and respiratory intensity (Gianfreda et al., 2005; Tripathi et al., 2007). Alkaline phosphatase enzymes (S-ALP) are believed to be mainly released by microorganisms (Nannipieri et al., 2011) and to be affected by plant species, microbial community composition, and rhizodeposition (Badri and Vivanco, 2009; Wasaki et al., 2018; Teixeira et al., 2019). Moreover, alkaline phosphatase enzymes are ubiquitous in soils, but their activity is somehow influenced by the quantity and quality of rhizodeposits (Ragot, 2016; Wasaki et al., 2018; Wu et al., 2018; Wei et al., 2019). In the current research, the activities of four soil enzymes all increased with continuous monocropping (Figure 1). Part of possible reasons were revealed by performing the linear fitting analysis of enzyme activity and soil physicochemical properties (Figure 9A) and ACE diversity index of microbial community (Figure 9B). Strong correlations were observed in S-UE and N (*R*^2^ = 0.36, *P* < 0.001), S-CAT and OC (*R*^2^ = 0.24, *P* = 0.008), S-SC and OC (*R*^2^ = 0.27, *P* = 0.005), and S-ALP and AP (*R*^2^ = 0.37, *P* < 0.001). The residual amount of nutrient elements differentiated due to the different utilization capacity of each cut chrysanthemum, and higher residual amounts always corresponded to higher enzyme activity. Still, the ACE index of fungal community increased with the increase of enzyme activity (Figures 9Be–h), which may indicate that cut chrysanthemum recruited more fungal taxa to increase enzyme production. The continuous increase of urease activity would be one of the reasons for the occurrence of replanting problems.

### Continuous Monocropping Caused Changes in the Bacterial Community Composition and a Decrease in the Abundance of Nutrient Element Conversion Genes

The richness and evenness of bacterial community did not change significantly during continuous monocropping of the four varieties (Figures 2A,B). However, variations in the abundance of some taxa occurred in the rhizosphere of cut chrysanthemum (Figures 3A,C, 4A). *Proteobacteria* is the dominant taxon in different geographical regions and soil types (Janssen, 2006), and members of this phylum play important roles in phylogenetic, ecological, and pathological values and energy metabolism (Bryant and Frigaard, 2006; Mukhopadhya et al., 2012). Root exudates and plant species play a key role in shaping the rhizosphere microbial community, which leads to genotype-specific communities of plants within the same soil type (Grayston et al., 1998; Marschner et al., 2004; Chaparro et al., 2014). The differences in the abundance of *Proteobacteria* may be primarily attributed to the varieties of cut chrysanthemum (Figure 3A). Based on previous studies, *Actinobacteria* is associated with pathogen antagonism (Mendes et al., 2011; Xiong et al., 2017), which is enriched in large amounts in suppressive soil and significantly decreased with the increase of monocropping time (Xiong et al., 2015). In our study, the relative abundance of *Actinobacteria* reduced significantly in the third year of monoculture. *Acidobacteria* is also a member of a relatively rich and prevalent soil bacterial community (Janssen, 2006), and it plays an important role in organic matter degradation and nutrient cycling (Eichorst et al., 2018). Fierer et al. (2007) showed that *Acidobacteria* had the highest abundance in soil with low resource availability. In general, continuous monocropping reduces soil nutrients and soil pH, which may explain the high abundance of *Acidobacteria* in replanted soil of cut chrysanthemum. In addition, *Bacteroidetes* participates in carbon and nitrogen metabolism, such as organic matter degradation and nitrite oxidation (Clemens, 2017; Han et al., 2017; Eichorst et al., 2018; Fang et al., 2018), and it has higher abundance in environments with high carbon content. The proportion of *Bacteroidetes* in replanted soil increased probably because more debris were produced in the roots of cut chrysanthemum. Other dominant phyla, *Acidobacteria* and *Chloroflexi*, also play an important role in organic matter degradation and nutrient cycling (Eichorst et al., 2018; Fang et al., 2018). For low classification levels, some species of *Pseudomonas* are involved in plant pathogen inhibition in many soils, and it serves as PGPR to reduce plant diseases (Liu et al., 1995; Kinkel et al., 2012). In the rhizosphere of cut chrysanthemum, the abundance of *Pseudomonas* decreased remarkably in the third year of continuous monocropping. Many *Bacillus* species, such as *Bacillus drentensis*, *Bacillus simplex*, and *Bacillus aryabhattai*, secrete fengycin, bacillaene, difficidin, and iturins by nutritional competition to antagonize pathogens such as *Rhizoctonia solani* and *Botrytis cinerea* (Ongena and Jacques, 2008; Jeong et al., 2012). The relative abundance of *Bacillus* did not change significantly in our study. *Rhizobium*, as a nitrogen fixer, was largely enriched in the rhizosphere of cut chrysanthemum, which may be attributed to the consumption of ammonium nitrogen in bulk soil. However, it should not be ignored that the species of *Pseudomonas* are not all beneficial; some are even plant pathogenic bacteria. In order to further improve the recognition rate of species and strains, the non-clustering algorithm of DADA3 may be better than that of UPARSE.

Changes in the composition of the bacterial community led to variations in metabolic genes, which were predicted by FAPROTAX based on 16S rRNA sequences. Continuous monocropping significantly reduced the abundance of genes related to nitrogen and sulfur metabolism in the rhizosphere of cut chrysanthemum (Figure 8A). Nitrogen is an important component of plant cell proteins, nucleic acids, chlorophyll, enzymes, etc., which can improve photosynthetic efficiency and increase crop yield and quality. As an element equally important to plants as NPK, sulfur is essential for the function of plant proteins, enzymes, and other physiologically active substances. The lack of sulfur element may lead to smaller plants and yellow leaves, which are symptoms of nitrogen deficiency, and ultimately result in the loss of crop yield and quality. With the increase of continuous cropping years, the reduction of the related metabolic genes will reduce the conversion rate of the rhizosphere nutrient of cut chrysanthemum and gradually cause a state of “element deficiency.” This state may contribute to the occurrence of reduced buds, smaller flowers, and increased diseases in cut chrysanthemum.

### Continuous Monocropping Significantly Increased the Number of Phytopathogenic Fungi in the Rhizosphere of Cut Chrysanthemum

During continuous monocropping, the richness of the rhizosphere fungal community significantly increased (Figure 2C). LEfSe showed significant changes in the abundance of multiple fungal taxa (Figure 4B). *Ascomycota* is the most dominant phylum in the rhizosphere of cut chrysanthemum. It contains many plant pathogens and is the main source of toxins that cause replanting diseases (Ecker et al., 2005; Li et al., 2014). It was significantly enriched in the rhizosphere of cut chrysanthemum with the increase of monocropping time. The third dominant phylum, *Basidiomycota*, has some species that are saprophytic, and many of its large saprophytic fungi cause wood rot. Nevertheless, other species can also promote the growth of plants by forming mycorrhizas with plants. In this study, *Basidiomycota* was significantly enriched in the rhizosphere of “variety 1.” At the genus level, *Mortierella* was involved in plant pathogen inhibition, which can inhibit the occurrence of the Chinese cabbage bulb disease and protect bananas against *Fusarium wilt* and root rot (Narisawa et al., 1998). It has higher abundance in vanilla suppressive soil, while it gradually accumulated in the rhizosphere of cut chrysanthemum with increasing planting time. *Humicola* also served as a biocontrol fungus (Sun et al., 2014), whose relative abundance increased significantly in the rhizosphere of cut chrysanthemum. Pathogenic fungi of some plant diseases, such as *Alternaria*, which can cause black spot in cabbage and *Alternaria Panax* in ginseng (Zhao et al., 1993; Ko et al., 2011), and *Fusarium*, which can cause wilt in chrysanthemums (Li et al., 2017), were enriched in the rhizosphere of cut chrysanthemum. *Rhizoctonia*, a pathogen that can cause blight in chrysanthemums, watermelons, etc. (Crater, 1992; Fehér, 1993), did not change significantly in our study.

To further reveal the changes of fungal community function, the FUNGuild method was used to divide fungal species into multiple guilds. A guild represents a more subdivided nutrition mode based on pathotroph, saprotroph, and symbiotroph, one of which is a plant pathogen. Guilds with significant changes were detected based on ANOVA. We found that some of the guilds belonging to plant pathogens were significantly enriched in the rhizosphere of cut chrysanthemum (Figure 8B). According to FUNGuild results, these guilds mostly belong to *Acremonium* (Hou et al., 2019), *Plectosphaerellaceae* (Giraldo and Crous, 2019), *Fusarium* (Ma et al., 2013), and *Cladosporium* (Carolina et al., 2021), which have been reported to be pathogens of certain crops. The pathogenic genus *Acremonium* (corresponding to Guild: Plant Pathogen D) has a relative abundance that even reached 50% in the rhizosphere of cut chrysanthemum (Top2 genus). Therefore, we speculated that the increased incidence of diseases during continuous monocropping of cut chrysanthemum was caused by not only *Fusarium* but also the joint action of multiple pathogens.

In the current research, four cultivars of cut chrysanthemum were selected to investigate the changes of the rhizosphere microbial community and soil physicochemical properties under different continuous cropping times. Continuous monoculture had less effect on the bacterial community, but it significantly reduced the number of bacteria that metabolized nitrogen and sulfur. Continuous cropping significantly increased the richness of rhizosphere fungi and decreased the abundance of chitin-lysis-related genes, prompting the rhizosphere of cut chrysanthemum to become a fungal community. The taxa largely enriched in rhizosphere belonged to phytopathogenic fungi, which may combine to disrupt the otherwise healthy microbial community and increase diseases in cut chrysanthemum. Continuous cropping did not always increase the number of pathogenic bacteria; some beneficial microorganisms were also enriched and promoted the increase of soil enzyme activity. But they seem unable to resist the trend of community degradation. In this study, ammonium nitrogen (AN) was one of the most important factors shaping microbial communities, and the variation of its content caused significant changes in bacterial and fungal communities. However, the total contribution rate of determined soil physicochemical properties to the community transformation was less than 50%, which suggested that there were other factors involved in shaping the rhizosphere microbial community. Allelochemicals are produced by leaves, roots, stems, etc., which have beneficial or adverse effects on other plants (Rice, 1984). Allelochemicals consist of OA, straight-chain alcohols, aldehydes or ketones, unsaturated lactones, fatty acids, etc. (Gross and Parthier, 1994; Einhellig, 1995; Rice, 1995; Seigler, 1996). If the donor and the recipient belong to the same species, this leads to intraspecific allelopathy, and the term used is autotoxicity (Singh et al., 2010). Autotoxicity is a major obstacle in continuous cropping under protected cultivation (Zhou et al., 2009), and it is also found in the cultivation of cut chrysanthemum. A variety of allelochemicals participate in the transformation of the composition and structure of the rhizosphere microbial community of cut chrysanthemum, and together with plant pathogens, they contribute to the replanting problem of cut chrysanthemum.

## Data Availability Statement

The original contributions presented in the study are included in the article/supplementary files, further inquiries can be directed to the corresponding authors.

## Author Contributions

ZR, XZ, and LL conceived and designed the experiments. TW, KY, and QM performed most of the experiments. TW and QM analyzed the data. TW wrote the manuscript. XJ and YZ helped to handle the plants. DK, ZW, and RP helped perform the analysis with constructive discussion. All authors read and approved the submitted version.

## Conflict of Interest

The authors declare that the research was conducted in the absence of any commercial or financial relationships that could be construed as a potential conflict of interest.

## Publisher’s Note

All claims expressed in this article are solely those of the authors and do not necessarily represent those of their affiliated organizations, or those of the publisher, the editors and the reviewers. Any product that may be evaluated in this article, or claim that may be made by its manufacturer, is not guaranteed or endorsed by the publisher.

## Footnotes

1
http://cran.r-project.org/
